# Derivation of a comprehensive semi-empirical proton RBE model from published experimental cell survival data collected in the PIDE database

**DOI:** 10.3389/fonc.2024.1415213

**Published:** 2024-11-27

**Authors:** Jian-Yue Jin, Jiankui Yuan, Xiaohang Qin, Yinghui Li, Huagang Yan, Nancy L. Oleinick, Min Yao, Quintin Pan, Feng-Ming (Spring) Kong, Mitchell Machtay

**Affiliations:** ^1^ School of Biomedical Engineering, Capital Medical University, Beijing, China; ^2^ Beijing Chest Hospital, Capital Medical University, Beijing, China; ^3^ Seidman Cancer Center, University Hospitals, Cleveland Medical Center, Cleveland, OH, United States; ^4^ Case Comprehensive Cancer Center, Case Western Reserve University School of Medicine, Cleveland, OH, United States; ^5^ Department of Radiation Oncology, Penn State University Cancer Institute, Hershey, PA, United States; ^6^ Department of Clinical Oncology, Hong Kong University Shenzhen Hospital, Shenzhen, China; ^7^ Department of Clinical Oncology, Queen Mary Hospital, Li Ka Shing Medical School, The University of Hong Kong, Hong Kong, Hong Kong SAR, China

**Keywords:** relative biological effectiveness (RBE), proton therapy, linear energy transfer (LET), multiple curve fitting (MCF), cell survival model

## Abstract

We aimed to develop a comprehensive proton relative biological effectiveness (RBE) model based on accumulated cell survival data in the literature. Our approach includes four major components: (1) Eligible cell survival data with various linear energy transfers (LETs) in the Particle Irradiation Data Ensemble (PIDE) database (72 datasets in four cell lines); (2) a cell survival model based on Poisson equation, with *α* and *β* defined as the ability to generate and repair damage, respectively, to replace the classic linear–quadratic model for fitting the cell survival data; (3) hypothetical linear relations of *α* and *β* on LET, or $\frac{\alpha \left(LET\right)}{\alpha_{x}}={\text{α}}_{\alpha }+b_{\alpha }\;*\;LET$ and $\frac{\beta \left(LET\right)}{\beta_{x}}={\text{α}}_{\beta }-b_{\beta }\;*\;LET$ ; and (4) a multi-curve fitting (MCF) approach to fit all cell survival data into the survival model and derive the *a_α_*, *b_α_*, *a_β_*, and *b_β_* values for each cell line. Dependences of these parameters on cell type were thus determined and finally a comprehensive RBE model was derived. MCF showed that (*a_α_*, *b_α_*, *a_β_*, *b_β_*) = (1.09, 0.0010, 0.96, 0.033), (1.10, 0.0015, 1.03, 0.023), (1.12, 0.0025, 0.99, 0.0085), and (1.17, 0.0025, 0.99, 0.013) for the four cell lines, respectively. Thus, *a_α_* = 1.12 ± 0.04, *b_α_* = 0.0019 ± 0.0008, *a_β_* = 0.99 ± 0.03, and *b_β_* = 0.013 ∗ *α_x_*, and approximately $\alpha ∼1.12*\alpha_{x}$ and $\beta =\left(0.99-0.013*\alpha_{x}*LET\right)*\beta_{x}$ . Consequently, a relatively reliable and comprehensive RBE model with dependence on LET, *α_x_*, *β_x_*, and dose per fraction was finally derived for potential clinical application.

## 1 Introduction

Proton therapy has a distinct dosimetric advantage over photon or electron radiotherapy due to the Bragg Peak (1, 2). However, this dosimetric advantage has not yet fully translated into corresponding clinical improvements for many disease sites. The poor understanding of relative biological effectiveness (RBE) of proton may be one of several important factors that have hindered the optimal use of this advanced technology, and consequently undermined the clinical outcome. A constant RBE = 1.1 has been used for proton therapy treatment planning despite the fact that RBE depends on many factors, including linear energy transfer (LET), radiation dose per fraction, and cell type (3, 4). An optimized dosimetric plan based on the constant RBE may not reflect the actual radiation effect and could turn out to be a poor plan in reality. A reliable RBE model is needed in proton therapy, especially in the era of intensity modulated proton therapy (4). Large amounts of effort have been devoted to this topic. Cell survival experiments under various LETs, doses, and cell types have been reported (5, 6) and many RBE models have been proposed (5–12). However, these models have not been adopted clinically by the proton therapy community.

One category of these models is the mechanism-inspired models, which include the saturable repair model, the Katz model, the local-effect model (LEM), the microdosimetric-kinetic model (MKM), and the repair–misrepair–fixation (LMF) model (7–12). Although these models may partially describe the complicated radiobiological process in different aspects, the underlying mechanism of proton radiobiology is not fully understood and described. In addition, these models have not fully utilized the large amount of experimental data in the literature. The other category includes those models mainly based on the experimental data (5–7). RBEs can be directly calculated from the experimental data, and RBE models depending only on the LET or dose can be simply determined. However, these models were often inconsistent among different studies and cell types, making their clinical application difficult. Theoretically, a comprehensive RBE model that depends on both LET and dose could be derived by a two-step fittings approach (6, 7), that is, first to fit each cell survival dataset with the linear–quadratic (LQ) model to derive its two parameters, *α_LQ_* and *β_LQ_*, for various LETs, and then to fit the *α_LQ_* and *β_LQ_* versus LET data with a hypothetical relation of *α_LQ_* and *β_LQ_* on LET (6, 7). However, because the LQ model is empirical, *α_LQ_* and *β_LQ_* are arbitrary fitting parameters that do not have a clear mechanistic relation with LET, the derived *α_LQ_* and *β_LQ_* values usually have large variations, and their dependence on LET is difficult to determine. Consequently, derivation of a comprehensive model using this two-step fittings approach was not satisfactory (6, 7).

This study used a different approach to overcome the problem. Firstly, we proposed a different cell survival model to replace the LQ model. As to be described in detail later on, the model was derived from Poisson equation (13), with two parameters, *α* and *β*, representing the ability of generating damage by radiation, and the ability of repairing the damage by cells, respectively, and we assumed that *α* linearly increases with increasing LET, and *β* decreases with increasing LET according to their definition. Secondly, we used a multi-curve fitting (MCF) approach (14) instead of the two-step fittings approach to directly derive the parameters that describe the dependence of *α* and *β* on LET. Specifically, we first presented the derivation of the new cell survival model, along with a test of the model’s fit to the experimental data; we then utilized the MCF approach to demonstrate the linear relations of *α* and *β* with LET and to derive their linear parameters; and finally we presented the derivation of the comprehensive RBE model from the experimental data. In addition, we found that the estimation of LET values in some reported experimental data in the literature might not be reliable, possibly due to the complicated composition of protons with different LETs (15) and not accounting for the significant increase of LET for high-LET protons when they passed through a cell (16).

## 2 Materials and methods

### 2.1 Derivation of the cell survival model

The derivation of the cell survival model was also described elsewhere (17, Qin et al.^1^). Statistically, radiation may generate different severities of DNA damage in different cells. We used the number of damage levels or unit damages to quantify the severity (we assume that one unit damage can equal to an arbitrary number of damage sites in a cell). Specifically, we defined *α* as the average number of unit damages that can be generated per unit dose. Thus, according to the Poisson equation (13), the probability of a cell having *n* (*n* = 0, 1, 2, 3…) unit damages is


(1) $$\begin{array}{c} \text{P}\left(\text{n}\right)=\frac{{\left(\alpha \text{D}\right)}^{\text{n}}}{\text{n}!}*{\text{e}}^{-\alpha \text{D}} \end{array}$$

where *D* is the radiation dose. We further defined *β* as the fraction of cells that can be repaired and survive when the cells have one unit damage, and the fraction of cells that can survive is *β^n^* when the cells have *n* unit damages (*n* = 0, 1, 2, 3…). Therefore, the overall survival fraction (SF) is the sum of repaired cells from cells with various unit damages. That is,


(2) $$\begin{array}{c} \text{SF}\left(\text{D}\right)=\left(1+\text{β}*\alpha \text{D}+{\text{β}}^{2}*\frac{{\left(\alpha \text{D}\right)}^{2}}{2}+\ldots +\beta^{\text{n}}*\frac{{\left(\alpha \text{D}\right)}^{\text{n}}}{\text{n}!}+\ldots \right)*{\text{e}}^{-\alpha \text{D}} \end{array}$$

We assumed that when a cell’s damage is very severe, or *n* is larger than a threshold number *m*, the damage is not repairable at all and the cell has 0% chance of survival. That is, when $n>m,\;\beta^{n}=0$ . Considering that when $m\to 0,\;SF\left(D\right)=e^{-\alpha D}$ , and when $m\to \infty ,\;\;SF\left(D\right)=e^{-\alpha \left(1-\beta \right)D}$ ; both approach to a linear model could not correctly describe the dose effect. Therefore, *m* should be a number not too small or too large. We found that when *m* = 3–6, all cell survival data could be well fitted by **Equation 2** (better than the LQ model), and further increasing *m* appeared to not improve the fitting (Qin et al.^1^). In this study, we used *m* = 5. Consequently, **Equation 2** becomes


(3) $$\begin{array}{c} S\text{F}\left(\text{D}\right)=\left(1+\alpha \beta \text{D}+\frac{{\text{α}}^{2}{\text{β}}^{2}{\text{D}}^{2}}{2}+\frac{{\text{α}}^{3}{\text{β}}^{3}{\text{D}}^{3}}{6}+\frac{{\text{α}}^{4}{\text{β}}^{4}{\text{D}}^{4}}{24}+\frac{{\text{α}}^{5}{\text{β}}^{5}{\text{D}}^{5}}{120}\right)*{\text{e}}^{-\alpha \text{D}} \end{array}$$

**Equation 3** can even be considered as a mechanistic model because it was derived from basic principles.

Because protons (and heavy ions) generate highly localized double-strand breaks or clustered DNA damages (CDDs), and increasing LET increases CDDs (18–21), the threshold number of damages may reduce with increasing LET. This is equivalent to *α* increasing with LET when *m* is fixed to 5. Similarly, because of the higher CDD density, the more difficult it is to repair the damages, such that *β* decreases with increasing LET. Therefore, *α* and *β* have a direct and mechanistic relation with LET. We could assume that this relation is linear or another simple form to determine the exact parameters in the relation by MCF with experimental data. This is the major advantage of this model over the LQ model. In addition, these parameters may directly relate to the cell characteristics. For example, parameter *α* may depend on the size, DNA folding structure, and the hypoxia status of the cell, and parameter *β* may depend on the genetic feature of the cell in DNA repair genes.

### 2.2 Particle Irradiation Data Ensemble database

We utilized published experimental cell survival data collected in the Particle Irradiation Data Ensemble (PIDE) database (5) for the study. The PIDE database was kindly provided by Dr. Thomas Friedrich. It consisted of *in vitro* cell survival experiment data of photon and ion irradiation from 115 publications (5). Among them, 34 publications had proton data in approximately 36 different cell lines.

### 2.3 Exclusion of unreliable experimental data

We found that the following experimental datasets were unreliable and were excluded from model-building: (1) Pristine proton datasets with LET > 33 keV (because high-LET data are prone to errors due to substantial increase of LET when protons travel through a monolayer of cells or a distance of air); (2) Spread-out Bragg Peak (SOBP) datasets with LET > 10 keV (because of significant amounts of high-LET protons in the SOBP beams and other reasons described in the Discussion section); and (3) datasets of two cell lines that were taken using a special high-throughput experimental device with protons irradiating from the back of a 96-well plate through a non-uniform plastic layer, which would induce large variation of LET, along with scattering protons from an LET-modulating jig into neighboring wells. The detailed reasoning for these exclusions is described in the Discussion section.

### 2.4 Exclusion of incomplete datasets

Many cell lines only have cell survival datasets of one, two, or three LET data points, and these data are not sufficient to derive reliable information for the cell lines and were excluded for the study. Some cell lines have datasets from different publications. However, some publications only have one LET dataset, or the cell survival datasets only have two to three dose points, or have very extremely large experimental variations in the cell survival datasets, or the study was performed on special cell cycles. These datasets were also excluded from the analysis.

### 2.5 Data included in the study

Finally, a total of 63 proton datasets in four cell lines (AG01522, U87, V79 and C3H10T1/2) were included in the study (**Table 1**). The data of AG01522 and U87 were from the same publication (22), which included six datasets for pristine protons, and six datasets for SOBP protons, for both AG01522 and U87 cells, respectively. These SOBP data were not excluded because they were used in an initial study to demonstrate that the data of protons on the distal edge of the SOBP were highly unreliable. The V79 has a total of 34 datasets in six publications (23–28), and the C3H10T1/2 has 5 datasets in one publication (29).

**Table 1:** Summary of experimental cell survival datasets used in the study.

Publication	Cell line	Beam type	No. of proton datasets	No. of photon datasets	LET (keV/µm)
Chaudhary (22)	AG01522^a^	Pristine	6	1	1.1–22.6
Chaudhary (22)	AG01522	SOBP	6	1	1.2–25.9
Chaudhary (22)	U87^b^	Pristine	6	1	1.1–22.6
Chaudhary (22)	U87	SOBP	6	1	1.2–25.9
Wouters15 (23)	V79^c^	SOBP	11	1	2.33–6.23
Wouters96 (24)	V79	SOBP	10	1	1.03–4.74
Folkard96 (25)	V79	Pristine	3	0*	10.1–27.6
Belli96 (26)	V79	Pristine	4	1	7.7–30.5
Prise90 (27)	V79	Pristine	3	0*	16.9–31
Folkard89 (28)	V79	Pristine	3	0*	17–32
Bettega (29)	C3H10T1/2^d^	Pristine	5	1	17–32

a AG01522 cell line: fibroblast of the foreskin, human.

b U87 cell line: malignant gliomas, human.

c V79 cell line: fibroblast morphology isolated from the lung of a male Chinese hamster.

d C3H10T1/2 cell line: exhibiting fibroblast morphology that was isolated from a line of C3H mouse embryo cells.

*Photon dataset was generated using the *α_LQ_* and *β_LQ_* data given in the publication.

### 2.6 Fitting experimental data to models

A build-in Solver program in Excel was used for fitting of experimental data with models. Because the survival fraction of cells usually ranges from 1 to 0.001, the root mean square percentage error (RMSPE) was used as an indicator of goodness of fitting, with a minimum of RMSPE corresponding to the best fitting. The RMSPE was defined as


(4) $$\begin{array}{c} \text{RMSPE}=\sqrt{\frac{1}{\text{n}}\sum_{\text{i}=1}^{\text{n}}{\left(\frac{{\text{P}}_{\text{i}}-{\text{O}}_{\text{i}}}{{\text{O}}_{\text{i}}}\right)}^{2}} \end{array}$$

where *n* is the number of experimental data points, *O_i_* is the experimentally measured survival fraction for each dose point, and *P_i_* is the corresponding expected survival fraction from the model. The data point at zero dose was not considered as a data point.

### 2.7 Testing the cell survival model

The cell survival model was tested by evaluating whether it could fit well with experimental cell survival data for the 63 proton and 8 photon datasets. Visual evaluation was first performed. Quantitative evaluations were accomplished by comparing the RMSPE of each dataset and the average RMSPE of all 71 datasets between our cell survival model and the LQ model.

### 2.8 MCF to determine the dependence of *α* and *β* on LET

We used *R_α_* and *R_β_* to represent *α* and *β*, which were defined as


(5) $$\begin{array}{c} {\text{R}}_{\text{α}}=\;\frac{{\text{α}}_{\text{p}}}{{\text{α}}_{\text{x}}}\;\text{and}\;{\text{R}}_{\text{β}}=\;\frac{{\text{β}}_{\text{p}}}{{\text{β}}_{\text{x}}} \end{array}$$

where *α_p_* and *β_p_* are *α* and *β* for proton beams with various LETs, and *α_x_* and *β_x_* are that for photons, respectively (x-rays or γ-rays).

We hypothesized that *R_α_* and *R_β_* were linear functions of LET when LET was not too large, that is:


(6a) $$\begin{array}{c} {\text{R}}_{\text{α}}={\text{a}}_{\text{α}}+{\text{b}}_{\text{α}}*\text{LET} \end{array}$$

(6b) $$\begin{array}{c} {\text{R}}_{\text{β}}={\text{a}}_{\text{β}}-{\text{b}}_{\text{β}}*\text{LET} \end{array}$$

where *a_α_*, *b_α_*, *a_β_*, and *b_β_* are parameters that may depend on cell type. Then, we used an MCF approach (14) to determine the *a_α_*, *b_α_*, *a_β_*, and *b_β_* values. The fitting variables *α* and *β* for multiple survival datasets at various LETs are replaced by four fitting variables *a_α_*, *b_α_*, *a_β_*, and *b_β_* using **Equation 6**, so that the best fitting values of the four variables can be directly determined by the MCF process to achieve a minimal sum of RMSPE for all datasets.

### 2.9 Evaluation of the MCF results

The MCF approach combines two-step fittings into one MCF process by assuming a perfect linearity in **Equation 6**. The price of this perfect linearity is that the fitting of individual survival dataset may be deteriorated from its best fitting. Although the sum of RMSPE can represent the overall goodness of fitting for MCF, it cannot reflect the deterioration of individual dataset fitting. Here, we define a deterioration index $Det=RMSPE/\left(RMSPE_{B}+0.05\right)$ to quantify the deterioration of fitting of each individual survival dataset by MCF, where *RMSPE* and *RMSPE*_B_ are the RMSPE for the MCF and best fitting, respectively, for the individual dataset, and the small number 0.05 is added to avoid a large *Det* when *RMSPE*_B_< 0.05. We found that when *Det* ≤ 1.25, the deterioration was almost undetectable visually; when *Det* ≤ 2, the deterioration was still small and generally well acceptable; however, when *Det* > 4, the deterioration was very large that it might indicate an outlier due to experimental errors or extremely large experimental uncertainty. When majority of datasets have a large *Det*, it may indicate that the linear relations do not hold.

### 2.10 Determination of the dependence of *α* and *β* on cell type

When evaluating the *a_α_*, *b_α_*, *a_β_*, and *b_β_* values for the four cell types, we found that *a_α_* and *a_β_* only slightly varied with cell type. In addition, *b_α_* was a small number having minimal impact on *α*. Therefore, for simplicity, we assumed that *a_α_*, *b_α_*, and *a_β_* were cell-independent. The average *a_α_*, *b_α_*, and *a_β_* values were calculated. New MCF was performed by considering *b_β_* as the only variable, and forcing *a_α_*, *b_α_*, and *a_β_* being the average values for each of the four cell types. New *b_β_* values (*b_β_^#^*) were determined and the dependence of *b_β_^#^* on cell type was derived by linear regressions of *b_β_^#^* with *α_x_* and other cell-type parameters such as *β_x_* and *α_x_* * *β_x_*.

### 2.11 Derivation of an RBE model

The RBE value for a given LET, dose (*D*), and cell type (represented by *α_x_* and *β_x_*) was determined by the following steps: (1) calculate the *SF(D)* for photon by using **Equation 3** with given *D*, *α_x_*, and *β_x_*; (2) calculate *α_p_* and *β_p_* using **Equations 5** and **6** for a given LET; (3) derive the *D_P_* (proton dose) by solving **Equation 3** for proton with given *D_P_*, *α_p_*, and *β_p_*, and *SF(D_P_)* = *SF(D)*; and (4) calculate RBE as *D*/*D_P_*. Because **Equation 3** is a relatively complicated function, an iterative computation process was used to solve the equation and derive the *D_P_* in step 5. Thus, the dependences of RBE on LET, *D*, *α_x_*, and *β_x_* were calculated and plotted.

## 3 Results

### 3.1 The cell survival model well fitted to all experimental data

**Figure 1:**
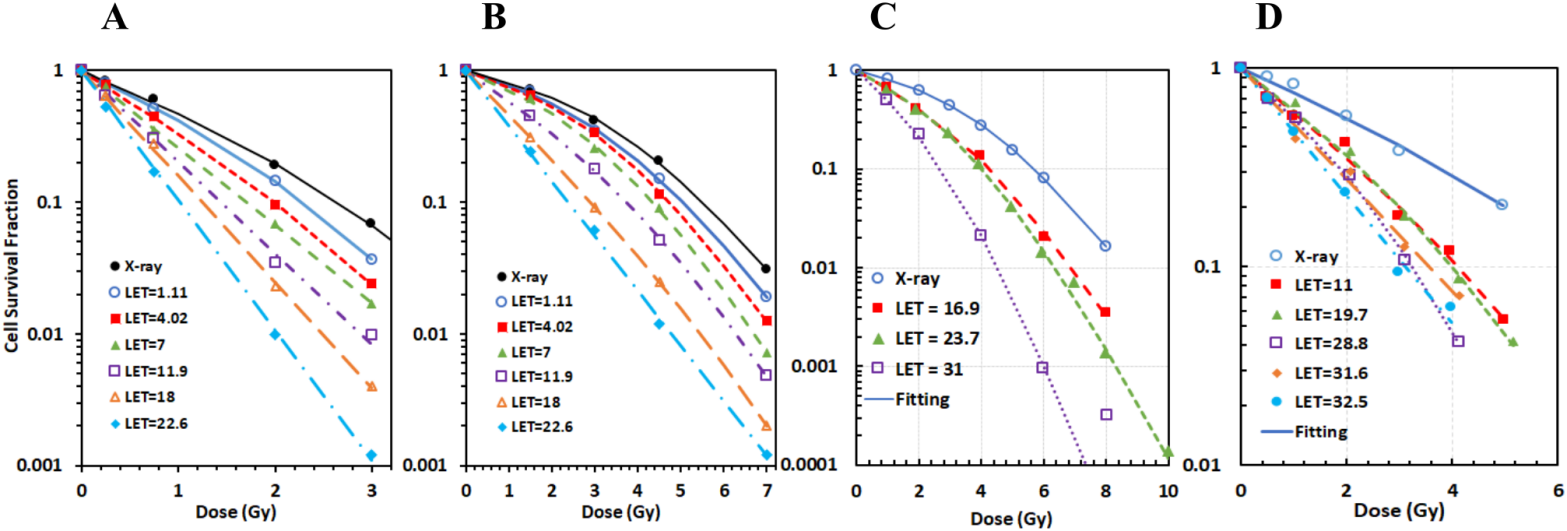
The new cell survival model fitted well with each experimental dataset for all four cell lines. Representative fittings of cell survival data for **(A)** AG01522, **(B)** U87, **(C)** V79, and **(D)** C3H10T1-2 cells.

All 63 proton and 8 photon cell survival datasets were well fitted with our cell survival model. **Figure 1** shows representatives of these fittings for the four cell lines, respectively. A quantitative evaluation showed that the average RMSPE was 6.6% ± 5.7% for the 71 datasets, with a range from 0.3% to 41%. In comparison, the average RMSPE was 6.6% ± 5.8% when the data were fitted with the LQ model, with a range from 1.3% to 44%. The two models had almost the exact same result. The large RMSPEs in some datasets were mainly due to the uncertainty of the experimental data rather than the fittingness of the model.

### 3.2 MCF showed linear relations of *α* and *β* on LET

**Figure 2:**
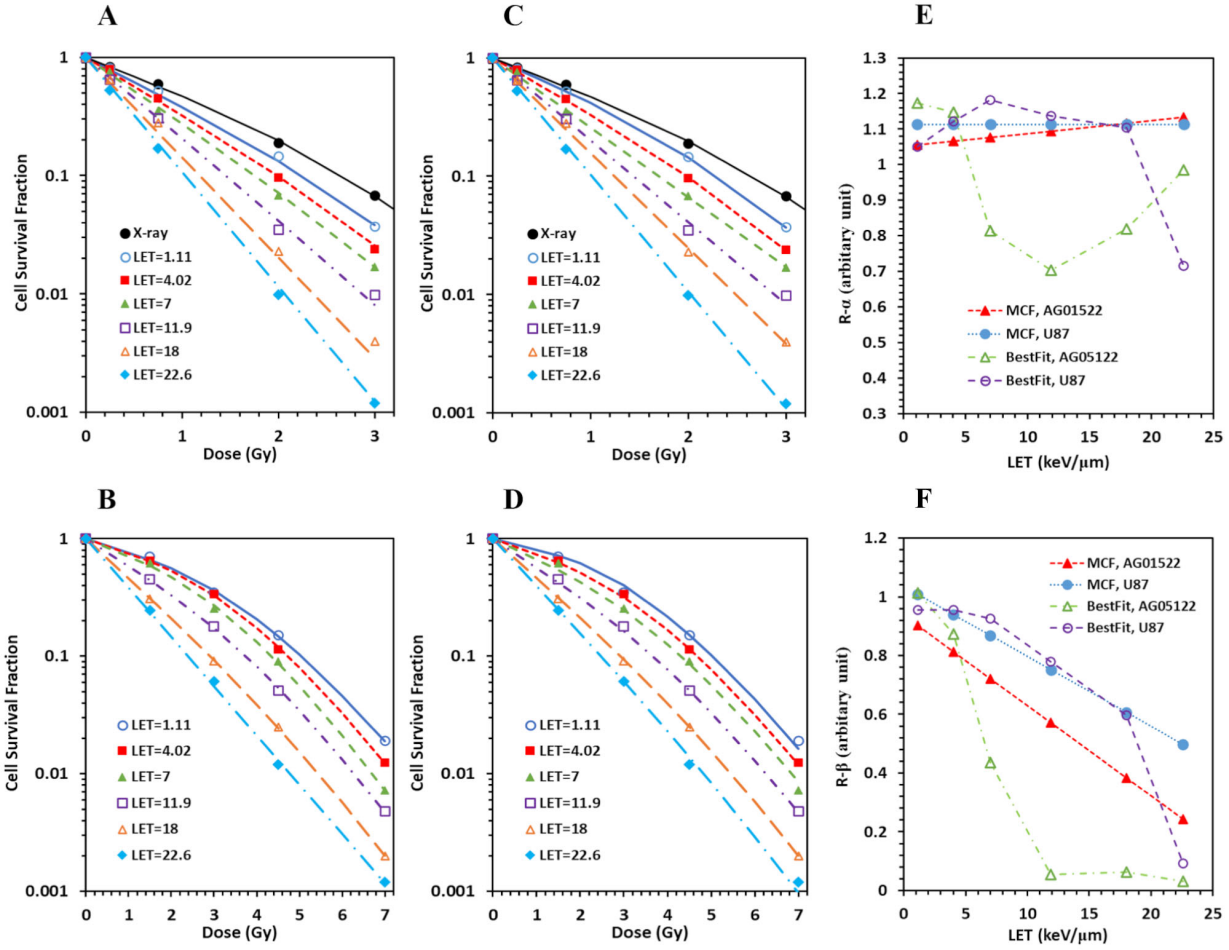
Multi-curve fitting (MCF) achieved minimal deterioration in cell survival data fittings with perfect linear relations of *α* and *β* on LET in comparison to conventional best fitting of individual cell survival data with irregular relations of *α* and *β* on LET for the pristine protons on both AG01522 and U87 cells. **(A)** Best fitting of individual cell survival dataset for AG 01522. **(B)** Best fitting for U87. **(C)** MCF of six cell survival datasets with various LETs for AG01522. **(D)** MCF for U87. **(E)** Comparison of the relation of *R_α_* on LET between MCF and best fitting. **(F)** Comparison of the relation of *R_β_* on LET between MCF and best fitting.

The MCF of the pristine proton datasets for two cell lines (AG01522 and U87 with LET varying from 1 to 25 keV/μm) showed almost invisible deteriorations by MCF (**Figures 2C, D**) in each dataset in comparison to the best fittings (**Figures 2A, B**) for both cell lines, suggesting that *α* and *β* are linearly related to LET (**Figures 2E, F**). The average RMSPE of the six datasets was 4.7% and 3.1% for the two cell lines, respectively. The maximal *Det* = 1.49, with majority of datasets having *Det* ≤ 1.25 for the 12 datasets. We also plotted the dependence of *α* and *β* on LET for the conventional two-step fittings approach. As shown in **Figures 2E, F**, *R_α_* and *R_β_* vary with LET in irregular patterns, suggesting that the relations of *α* and *β* on LET are difficult to derive using the two-step fittings approach.

### 3.3 Data for protons on the distal edge of SOBP were not reliable

**Figure 3:**
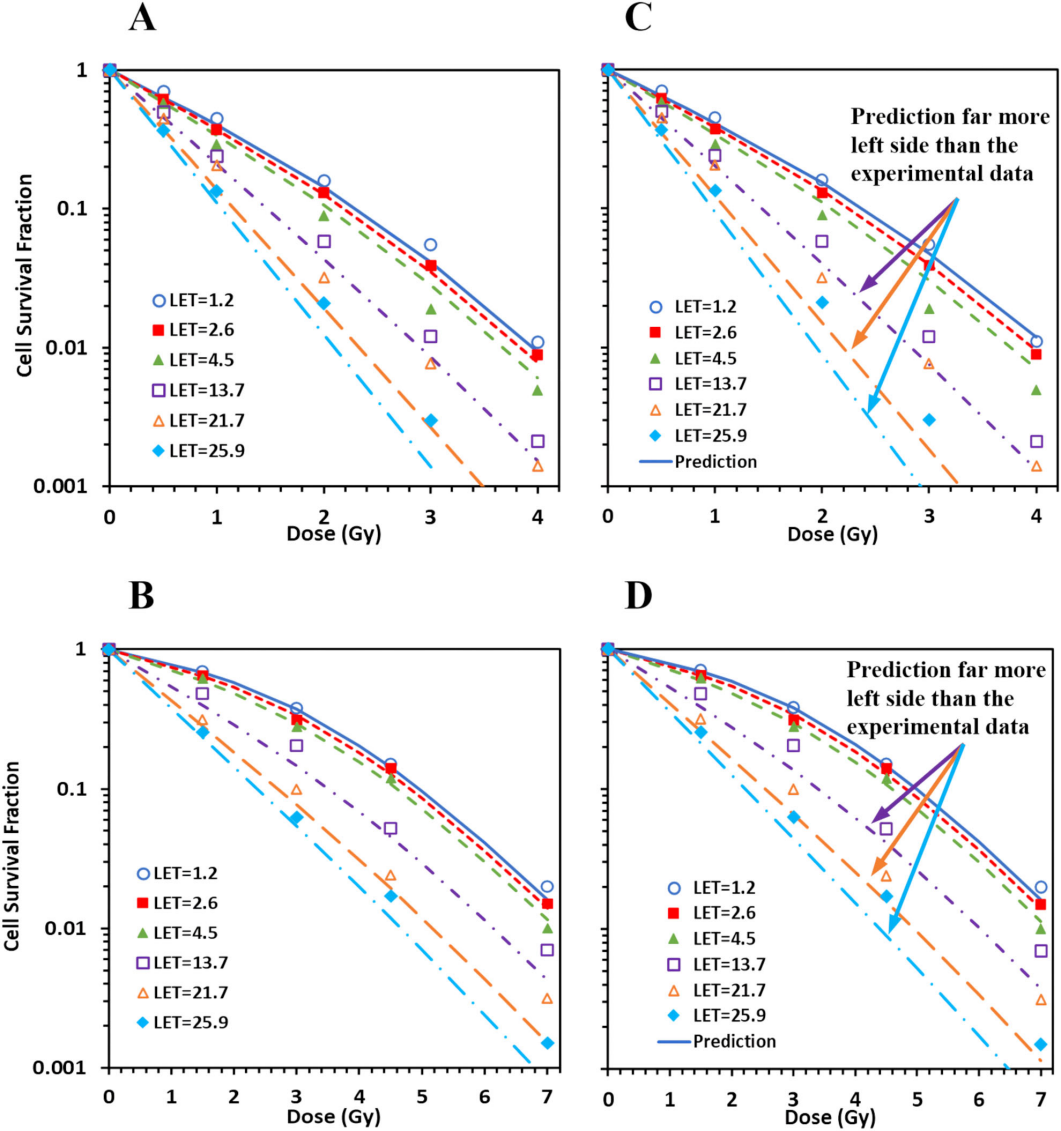
Spread-out Bragg peak (SOBP) proton data do not consist with pristine proton data. **(A, B)** When MCF was performed for combined pristine and SOBP data, large deteriorations (deterioration index ≥ 4) were induced in three datasets of protons on the distal edge of the SOBP (corresponding LET = 13.7, 21.7, and 25.9) for AG01522 **(A)**, with much smaller deteriorations for the pristine proton data (figure not show here). Similar deteriorations were induced for U87 **(B)**. **(C, D)** When the linear relation derived from the pristine data were applied to the SOBP data, the predicted survival curves showed consistent deviations from the experimental data for the three outliers for both AG 01522 **(C)** and U87 **(D)** cells.

MCF for combined pristine and SOBP data for both AG01533 (**Figure 3A**) and U87 (**Figure 3B**) cells showed large deteriorations. Det was 4.0, 4.0, and 4.5, respectively, for the three datasets of protons on the distal edge of the SOBP for the AG01522 cells (corresponding to LET = 13.7, 21.7, and 25.9 keV/µm, respectively) (**Figure 3A**), suggesting some errors in the three datasets. When the three datasets were excluded from MCF, the fitting results were greatly improved, with maximal *Det* = 1.8 and 1.5 for AG01522 and U87 cells, respectively. Moreover, when we applied the linear relation derived from the pristine data into the SOBP data, the predicted survival curves showed consistent deviations from the experimental data for the three datasets for both cell lines (**Figures 3C, D**). The predicted survival curves were much more in the left side than the corresponding experimental ones, suggesting that these errors were systematic errors, and most likely came from incorrect estimation of LETs.

### 3.4 Derivation of parameters *a_α_*, *b_α_*, *a_β_*, and *b_β_*

**Figure 4:**
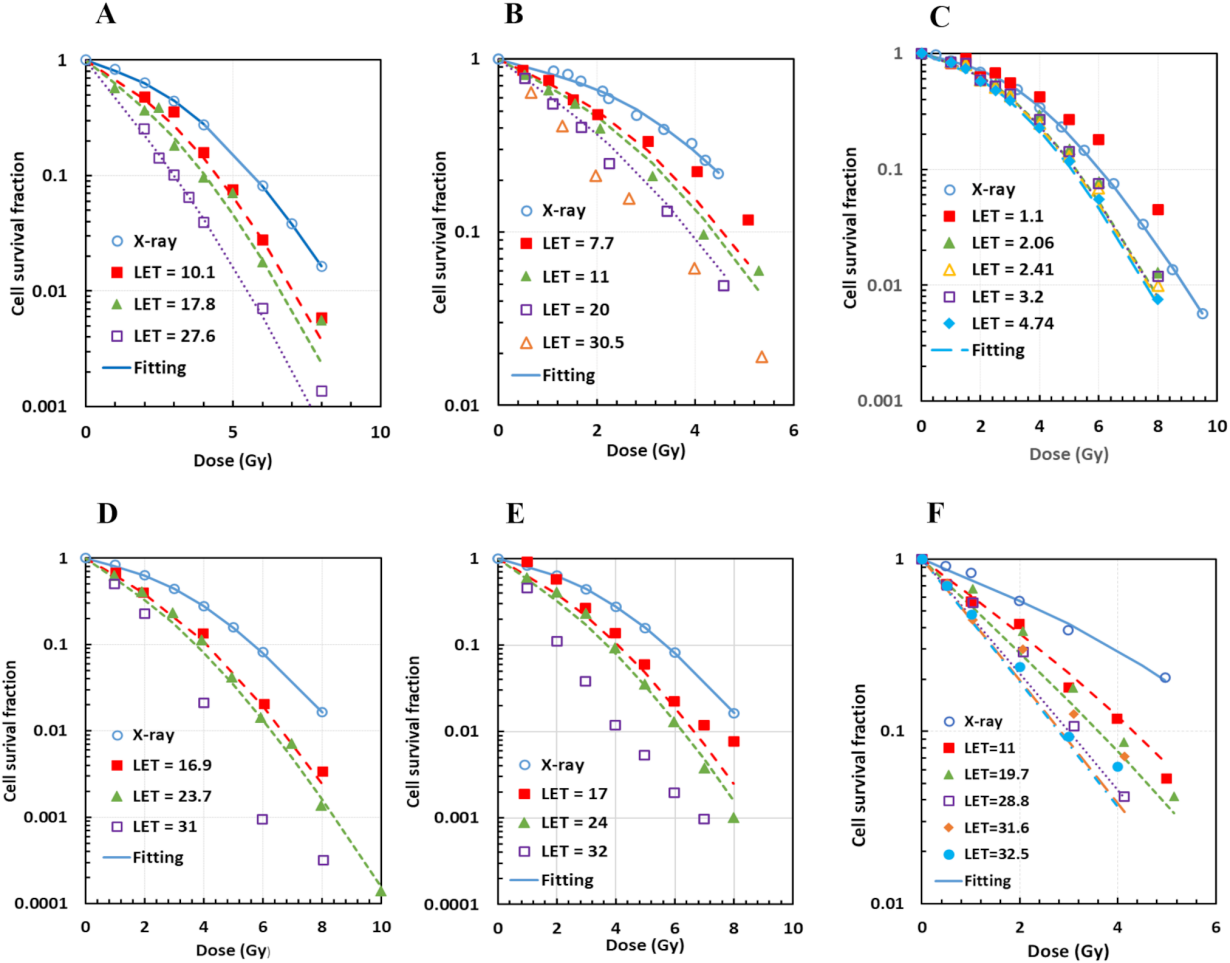
Acceptable multi-curve fitting (MCF) results for V79 cells with datasets from six different publications after exclusion of outliers, and for C3H10T1/2 cells from one publication. **(A)** Two datasets for V79 from Folkard96; **(B)** 4 datasets for V79 from Belli98, with one outlier (LET = 30.5 keV/µm) excluded; **(C)** 21 datasets for V79 from Wouters15 and Wouters96, with two outliers (LET = 1.03 and 1.1 keV/µm) excluded (not all datasets were shown); **(D)** 3 datasets for V79 from Prise90, with one outlier (LET = 31 keV/µm) excluded; **(E)** 3 datasets for V79 from Folkard89, with one outlier (LET = 32 keV/µm) excluded; **(F)** MCF for C3H10T1/2.

From MCF, we had (*a_α_, b_α_, a_β_, b_β_*) = (1.09, 0.0010, 0.96, 0.033) and (1.10, 0.0015, 1.03, 0.023) for AG01522 and U87 cells, respectively. MCF for V79 cells, which had 34 datasets from six publications, showed high deterioration, with maximal *Det* = 5.0 and six datasets having *Det* > 3. These deteriorations were mainly due to two factors: (1) two datasets with protons likely on the front edge of an SOBP (corresponding LET = 1.03 and 1.1 keV/µm) and (2) three datasets of pristine protons with LET ≥ 30 keV/µm. When the five datasets were excluded from the MCF, the fittings were greatly improved, with the maximal *Det* = 2.6 and majority of datasets having *Det<* 1.5 (**Figures 4A–E**). Thus, we had (*a_α_*, *b_α_*, *a_β_* and *b_β_*) = (1.12, 0.0025, 0.99, 0.0085) for V79 cells. The MCF result was acceptable to C3H10T1-2 cells (**Figure 4F**), with maximal *Det* = 2.3, and (*a_α_*, *b_α_*, *a_β_* and *b_β_*) = (1.17, 0.0025, 0.99, 0.013). Consequently, the *a_α_*, *b_α_*, *a_β_*, and *b_β_* values for all four cell lines were determined and are summarized in **Table 2**.

**Table 2:** List of the *a_α_*, *b_α_*, *a_β_*, and *b_β_* values from MCF, and the new *b_β_* (*b_β_^#^*) from single-variable MCF and the average *α_x_* for all four cell lines.

Cell line	*a_α_*	*b_α_*	*a_β_*	*b_β_*	*b_β_^#^*	*α_x_*
AG01522	1.09	0.001	0.96	0.033	0.033	2.34
U87	1.10	0.0015	1.03	0.023	0.020	1.45
V79	1.12	0.0025	0.99	0.0085	0.010	1.32
C3H10T1/2	1.17	0.0025	0.99	0.013	0.014	1.18

### 3.5 Derivation of the comprehensive RBE model

From **Table 2**, we noted that *a_α_* and *a_β_* slightly varied with cell type, and *b_α_* was a small number having a minimal impact on *α*. Thus, for simplicity, we assumed that *a_α_*, *b_α_*, and *a_β_* were cell-independent and they were determined as the average of the four cell lines. Therefore, *a_α_* = 1.12 ± 0.04, *b_α_* = 0.0019 ± 0.0008 (which might be considered as 0), and *a_β_* = 0.99 ± 0.03. A new single-variable MCF with *b_β_* as the only variable, and *a_α_*, *b_α_*, and *a_β_* being the above constants was performed for each cell line, and results were reasonably acceptable, with maximal *Det* = 1.9, 2.9, 2.8, and 2.6 for the AG01522, U87, V79, and C3H10T1-2 cells, respectively. The new cell-dependent *b_β_* was determined as *b_β_^#^* = 0.033, 0.019, 0.010, and 0.014 correspondingly.

**Figure 5:**
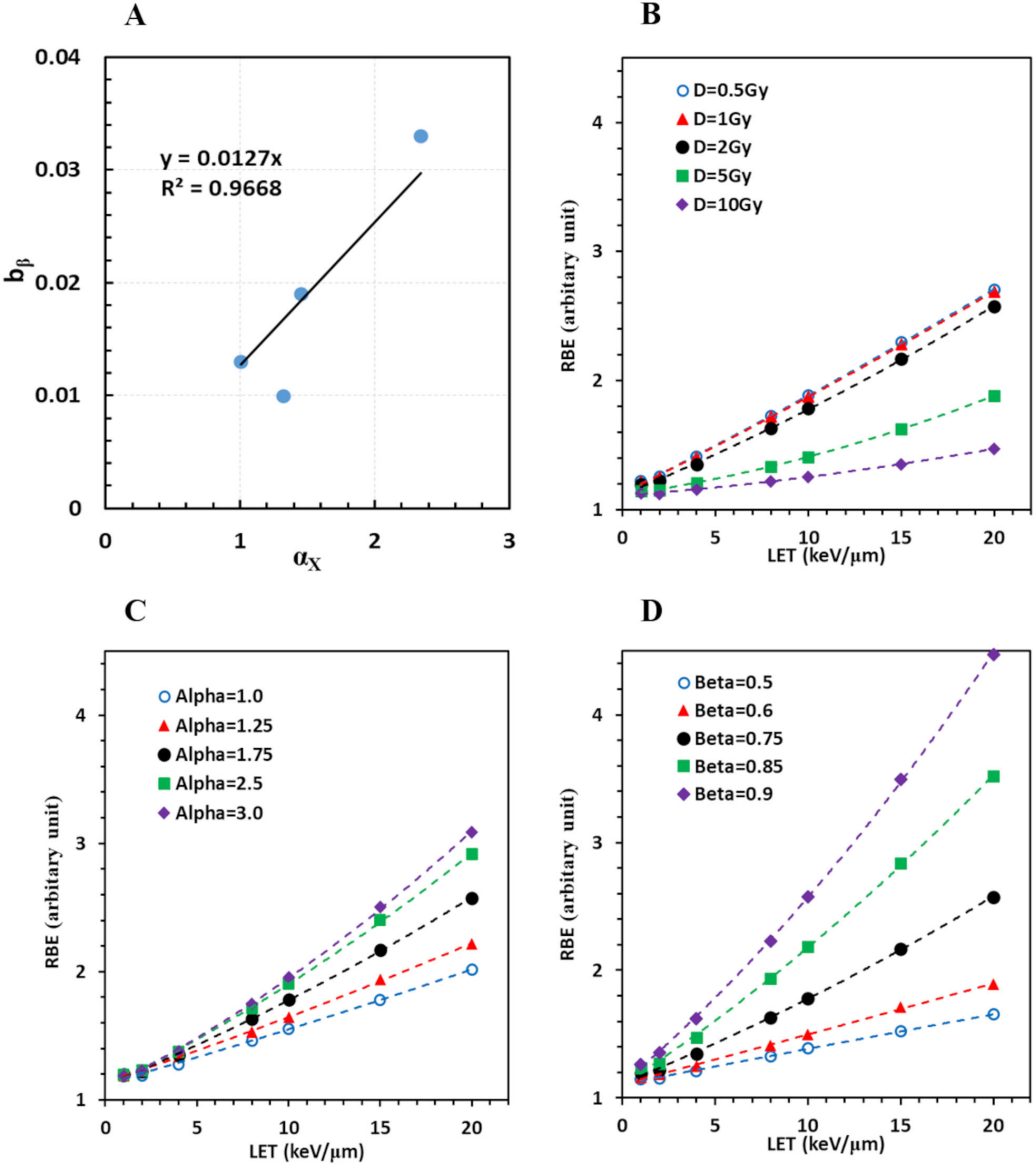
Derivation of a comprehensive RBE model. **(A)** Linear regression of *b_β_* on cell type represented by *α_x_*; **(B)** RBE increases with LET for various doses when *α_x_* = 1.75, *β_x_* = 0.75; **(C)** for various *α_x_* when dose = 2Gy, *β_x_* = 0.75; **(D)** for various *β_x_* when dose = 2Gy, *α_x_* = 1.75.


We found that *b_β_^#^* was linearly correlated with *α_x_* (**Figure 5A**) with a relation of

(7) $$\begin{array}{c} {\text{b}}_{\text{β}}^{\#}\left({\text{α}}_{\text{x}}\right)=0.013*{\text{α}}_{\text{x}} \end{array}$$

Thus, **Equation 6** became


(8a) $$\begin{array}{c} \text{α}=\left(1.12+0.0019*\text{LET}\right)*{\text{α}}_{\text{x}}∼1.12*{\text{α}}_{\text{x}} \end{array}$$

(8b) $$\begin{array}{c} \text{β}=\left(0.99-0.013*{\text{α}}_{\text{x}}*\text{LET}\right)*{\text{β}}_{\text{x}} \end{array}$$

Therefore, all eligible cell survival data in this study were reasonably modeled with **Equations 3** and **8**. Consequently, RBE values were computed given any cell line represented by *α_x_* and *β_x_*, under irradiation of any protons with a given LET, and at any dose per fraction. The RBE model expressed as the dependence of RBE on LET at various dose, *α_x_*, and *β_x_* values is shown in **Figures 5B–D**, respectively.

## 4 Discussion

Using an approach including a novel cell survival model, hypothetical linear relations of its parameters with LET, and an MCF method to fit multiple datasets of various LETs into the model, we have derived a semi-empirical RBE model based on all eligible proton cell survival datasets collected in the PIDE database (5). This RBE model is significant because it is able to predict RBE values for any cell lines represented by *α_x_* and *β_x_* for any given LETs and doses per fraction. In addition, the study has demonstrated that the approach is able to identify outlying datasets with large experimental errors. These outliers include datasets of SOBP protons on its distal and front edges, and pristine protons with LET > 30 keV/µm. Identifying these outliers and other potential outliers is significant because this may explain the inconsistency of many RBE models in literature, exclude those unreliable datasets to ensure a more accurate and reliable RBE model, and provide guidelines and knowledge for future RBE experiments. The ability to identify outliers also indicates that our cell survival model and linear relations reflect the true radiobiology of proton irradiation on cells.

The three SOBP datasets with LET > 10 keV/µm, which were likely on the distal edge of the SOBP plateau, showed significant and consistent deviations from the linear relations of *α* and *β* on LET for both AG01522 and U87 cells. These suggest a systematic error in the three datasets. The two SOBP datasets with LET = 1.03 and 1.1 keV/µm for V79 cells, which were likely on the front edge of the SOBP, were also outliers in MCF. In addition, those pristine protons with LET > 30 keV/µm for V79 cells also appeared to have large deviations. We speculated that these deviations were mainly due to incorrect computation of the LET or overlooking the substantial LET increase for high-LET protons passing through a monolayer of cells. It was reported that a proton facility miscalculated the 1-cm air gap with a 1-mm gap (30). Consequently, data published before 1991 from the facility had to be corrected. An LET of 31.6 was actually 64.8 keV/µm after correction, and 28.4 corresponded to 44.1 keV/µm. On the other hand, the miscalculation only changed an LET of 10.5 to 10.8, and 17.5 to 19.1 kV/µm (30), suggesting that high-LET data are prone to errors. It was also reported that the thickness of cells attached in mylar film for proton experiments was 6.1 and 2.9 µm for V79 and C3H10T1-2 cells, respectively (16). The thickness increases the actual LET by 15% and 6% for V79 and C3H10T1-2 cells when the nominal LET = 25.2 keV/µm, and by 29% and 10% when the nominal; LET = 30.7 keV/µm, respectively (16). These data well explained why the data points with LET > 30 keV/µm were outliers in V79 cells, but were relatively acceptable in C3H101-2 cells in our study. The SOBP beam is composed of protons with various LETs, with a large percentage of high-LET protons when its average LET is >10 keV/µm. Thus, the effect of increasing LET when protons pass through a cell could significantly and complicatedly change the nominal LET. In addition, other factors may also contribute to the deviations in SOBP. These include (1) dose calibration uncertainty due to the sharp dose gradient on the distal or front edge of the SOBP: a dosimeter position uncertainty, a limited depth resolution of the dosimeter, and a proton range uncertainty would all greatly affect the accuracy of dose calibration; (2) uncertainty of LET in Monte Carlo simulation, because SOBP protons have much more complicated energy spectrums, and a small deviation of beam parameters used in simulation may be amplified in SOBP beams.

The ability to identify outliers with large experimental errors also suggests that our models, including **Equations 3** and **6**, correctly describe the radiobiology of proton irradiation on cells. The definition of *α* as the ability to generate damages suggests that *α* may depend on the size of cell nucleus, DNA structure packing and folding (31), and the hypoxia status of the cells (32). Similarly, the definition of *β* as the damage repair capability suggests that *β* may depend on the genetic feature of the DNA repair genes (33, 34). Therefore, *α_x_* and *β_x_* may be directly determined using a model based on the cell’s morphology/structure and genetic feature. In fact, we found that *α_x_* was indeed proportional to the cell diameters in a different study. The ability to derive *α_x_* and *β_x_* by both a cell property-based model and experimental cell survival data may help to determine reliable *α_x_* and *β_x_* values for an accurate RBE model. For example, the DLD1 and HCT116 cells are similar human tumor cells (same morphology) with the only difference in p53 gene (35). We would expect similar *α_x_* and different *β_x_* values for the two cell lines. Thus, experimental data for the two cell lines with different LETs would provide valuable information and new perspective for the RBE model.

RBE appeared to increase with LET more rapidly with a smaller dose/fraction, a larger *α_x_*, and a larger *β_x_*, with *β_x_* being the most important factor among them. Interestingly, RBE was only slightly increased when the dose was further reduced from 1 to 0.5 Gy/fraction, suggesting that very low-dose distribution with high LET outside the target would not further enhance the effective dose and harm the normal tissue. Typical RBE was approximately 1.5–2 when LET~10 keV/μm, and increased to 2–3 when LET ~20 keV/μm, under the condition of *α_x_* = 1.5–2.5, *β_x_* = 0.7–0.8, and a dose of 2 Gy/fraction. Because the largest LET is ~83 keV/µm for protons (36), the largest RBE can reach to ~12.7 when *α_x_* = 2.5, *β_x_* = 0.9, and a dose of 0.5 Gy/fraction, assuming our model can extrapolate to higher LETs. This means that an end of range spot with a physical dose of 0.5 Gy/fraction may have an effective dose of 6.4 Gy/fraction. Fortunately, this is a very small volume, which may induce limited functional damage to the normal structure.

Although this study demonstrated a great potential in developing a comprehensive and reliable proton RBE model, the following are required before its clinical application: (1) perform more cell survival experiments to accumulate more reliable data for validation of the RBE model, especially data with human normal cells and human tumor cells to validate and improve the cell-dependent relations; (2) build an *α_x_* and *β_x_* library for various human tumor cells and human normal tissue cells, or derive models to convert *α_LQ_* and *β_LQ_* into *α_x_* and *β_x_*; and (3) apply the RBE models to patients who have been treated with proton therapy, evaluate the change of effective doses to the tumor and various organs, correlate the doses with clinical outcome of these patients, and finally determine whether and how the RBE model can be safely implemented clinically.

## 5 Conclusion

Using a different cell survival model from the classic LQ model, we have developed a proton RBE model based on all eligible experimental cell survival data collected in the PIDE database. We have also identified protons on the distal and front edges of a SOBP and pristine protons with LET > 30 keV/µm to be outliers with large experimental errors. The potential causes of these errors are well explained by physics principles, suggesting the reliability of this RBE model and its great potential for clinical application.

## Data Availability

Publicly available datasets were analyzed in this study. This data can be found here: https://academic.oup.com/jrr/article/54/3/494/973718. The interested researchers can contact Dr. Thomas for the dataset.

## Footnotes

1 Qin XH, Li YH and Jin JY. A quasi-mechanistic cell survival model to replace linear-quadratic model, unpublished data.
